# Nausea and Dyspnea on Exertion: Left Ventricular Free-wall Rupture

**DOI:** 10.5811/cpcem.2021.4.50265

**Published:** 2021-12-16

**Authors:** Juliana Wilson, Matthew Mendes, Marian E. Betz, Juan Galindez Mingo

**Affiliations:** * University of Colorado Denver – Anschutz Medical Campus, Department of Emergency Medicine, Aurora, Colorado; † Denver Health Medical Center, Department of Emergency Medicine, Denver, Colorado; ‡ University of Colorado School of Medicine, Aurora, Colorado

**Keywords:** Left ventricular free-wall rupture, POCUS, acute myocardial infarction

## Abstract

**Case Presentation:**

A 53-year-old female presented to the emergency department with three days of nausea and dyspnea on exertion after using methamphetamine. Initial electrocardiogram revealed an ST-elevation myocardial infarction. While awaiting transfer to the cardiac catheterization lab the patient suffered a witnessed cardiac arrest. During resuscitative efforts an enlarging pericardial effusion on point-of-care ultrasound led to the detection of a left ventricular free-wall rupture (LVFWR). This case illustrates the progression of a left ventricular free-wall rupture using point-of-care ultrasound.

**Discussion:**

Left ventricular free-wall rupture has a low incidence rate in the setting of an acute myocardial infarction. Ultrasonography is the tool of choice for detecting a LVFWR.

## CASE PRESENTATION

**Image 1:**
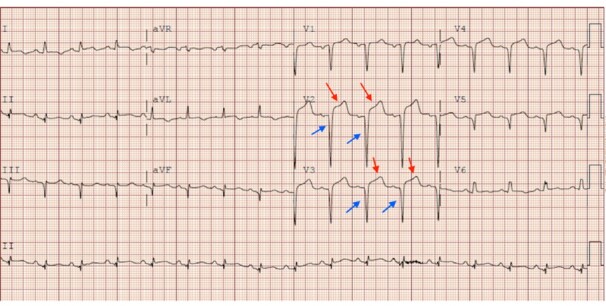
Electrocardiogram from initial workup demonstrating ST-segment elevations (red arrows) and Q waves (blue arrows).

**Image 2:**
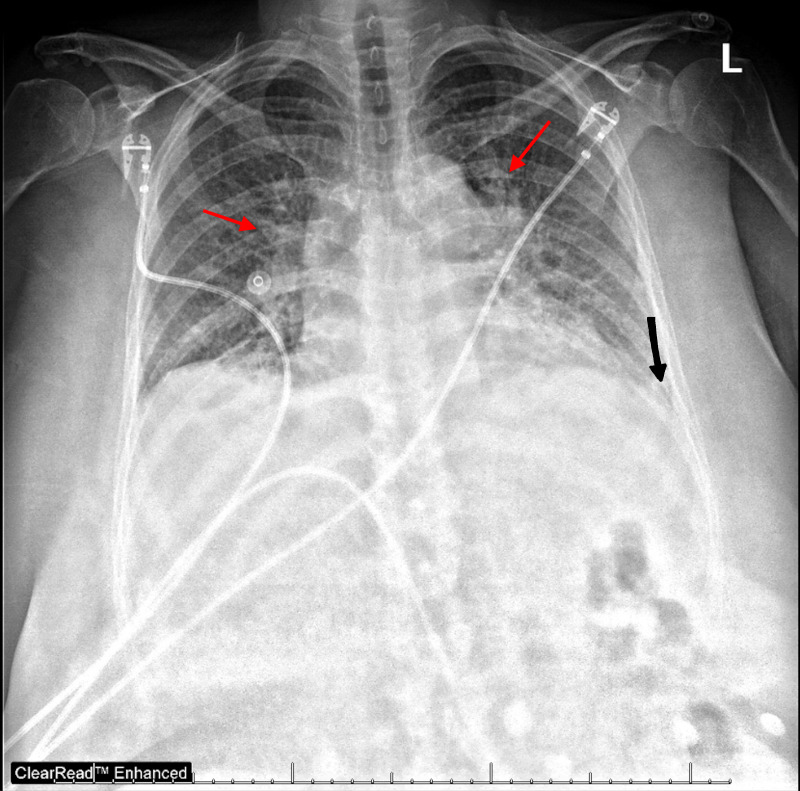
Chest radiograph showing pulmonary edema (red arrows) and small pleural effusions (black arrow).

A 53-year-old female with a past medical history of diabetes mellitus, hypertension, and substance use disorder presented to the emergency department with three days of nausea, dyspnea on exertion, and orthopnea. The patient’s initial vital signs showed a heart rate of 104 beats per minute, blood pressure of 193/109 millimeters of mercury, respiratory rate of 18 breaths per minute, and pulse oximetry of 94% oxygen saturation on room air. Her initial workup and evaluation demonstrated ST-segment elevations and Q waves on electrocardiogram (ECG) (Image 1). The cardiac catheterization laboratory was immediately activated upon review of the ECG. Chest radiograph (Image 2) was obtained indicating pulmonary edema and small pleural effusions.

An initial point-of-care ultrasound (POCUS) (Video 1) showed evidence of a very small pericardial effusion. The patient was initially hesitant to undergo cardiac catheterization but 30 minutes after arrival agreed to intervention. Prior to transfer to the catherization laboratory, she suffered a cardiac arrest. Cardiopulmonary resuscitation and advanced cardiac life support were initiated. During resuscitative efforts POCUS was obtained, which showed a large pericardial effusion with concern for a mechanical complication of acute myocardial infarction (MI). Left ventricular free-wall rupture (LVFWR) was the most concerning given the enlarging pericardial effusion (Video 2). Emergent pericardiocentesis via the subxiphoid approach was performed and 80 milliliters of bloody fluid was obtained. Repeat POCUS did not show any residual pericardial effusion following pericardiocentesis. Despite pericardiocentesis and resuscitative efforts, the patient did not survive.

## DISCUSSION

Diagnosis of LVFWR with hemopericardium following MI was confirmed through autopsy. A number of risk factors including diabetes mellitus, hypertension, and methamphetamine use could have contributed to a subacute MI (three days prior) resulting in LVFWR. Autopsy revealed the patient suffered from a 90% occluded left anterior descending artery. Left ventricular free-wall rupture will typically occur within five days of an MI due to vulnerable necrotic tissue.^1^ Due to coronavirus disease 2019-related factors such as fear of virus contraction, patients may be more likely to present later to the hospital, leading to higher incidence of post-MI complications.^2^ The incidence rate of LVFWR in acute MI is 2.2%.^3^ The use of methamphetamine can also increase the risk of developing atherosclerotic plaque and acute coronary vasospasm.^4^ Ultrasonography is indicated as the primary diagnostic tool if a patient is suspected of suffering from a LVFWR.^5^ Management of a LVFWR includes pericardiocentesis and positive inotropic agents along with emergent surgical repair.^5^

**CPC-EM Capsule**

What do we already know about this clinical entity?

*A Left ventricular free wall rupture (LVFWR) can occur due to weakening of the myocardium following a myocardial infarction. Ultrasonography is the primary tool used to diagnose LVFWR*.

What is the major impact of the image(s)?

*The echocardiograms presented in this case demonstrate the progression of a left ventricular free wall rupture leading to a large pericardial effusion*.

How might this improve emergency medicine practice?

*Recognizing patients at risk for LVFWR and identifying a pericardial effusion and myocardial rupture through ultrasonography can lead to prompt diagnosis and improved patient outcomes*.

## Supplementary Information

Video 1 Point-of-care ultrasound (parasternal short axis) prior to cardiac arrest showing a small pericardial effusion (black arrowheads) and a small myocardium rupture (white arrow).

Video 2 Point-of-care ultrasound during resuscitative efforts showing a large pericardial effusion (white arrows).
